# Tryptophan Derivatives by *Saccharomyces cerevisiae* EC1118: Evaluation, Optimization, and Production in a Soybean-Based Medium

**DOI:** 10.3390/ijms22010472

**Published:** 2021-01-05

**Authors:** Michele Dei Cas, Ileana Vigentini, Sara Vitalini, Antonella Laganaro, Marcello Iriti, Rita Paroni, Roberto Foschino

**Affiliations:** 1 Department of Health Sciences, Università degli Studi di Milano, 20142 Milan, Italy; michele.deicas@unimi.it (M.D.C.); rita.paroni@unimi.it (R.P.); 2 Department of Food, Environmental and Nutritional Sciences, Università degli Studi di Milano, Via G. Celoria 2, 20133 Milan, Italy; antonella.laganaro@gmail.com (A.L.); roberto.foschino@unimi.it (R.F.); 3 Phytochem Lab, Department of Agricultural and Environmental Sciences, Center for Studies on Bioispired Agro-Environmental Technology (BAT Center), National Interuniversity Consortium of Materials Science and Technology, Università degli Studi di Milano, 20133 Milan, Italy; sara.vitalini@unimi.it (S.V.); marcello.iriti@unimi.it (M.I.)

**Keywords:** kynurenine pathway, MEL biosynthesis, *Saccharomyces cerevisiae*, yeast, tryptophan extraction, LC-MS/MS, soybean

## Abstract

Given the pharmacological properti es and the potential role of kynurenic acid (KYNA) in human physiology and the pleiotropic activity of the neurohormone melatonin (MEL) involved in physiological and immunological functions and as regulator of antioxidant enzymes, this study aimed at evaluating the capability of *Saccharomyces cerevisiae* EC1118 to release tryptophan derivatives (dTRPs) from the kynurenine (KYN) and melatonin pathways. The setting up of the spectroscopic and chromatographic conditions for the quantification of the dTRPs in LC-MS/MS system, the optimization of dTRPs’ production in fermentative and whole-cell biotransformation approaches and the production of dTRPs in a soybean-based cultural medium naturally enriched in tryptophan, as a case of study, were included in the experimental plan. Variable amounts of dTRPs, with a prevalence of metabolites of the KYN pathway, were detected. The LC-MS/MS analysis showed that the compound synthesized at highest concentration is KYNA that reached 9.146 ± 0.585 mg/L in fermentation trials in a chemically defined medium at 400 mg/L TRP. Further experiments in a soybean-based medium confirm KYNA as the main dTRPs, whereas the other dTRPs reached very lower concentrations. While detectable quantities of melatonin were never observed, two MEL isomers were successfully measured in laboratory media.

## 1. Introduction

L-tryptophan (L-TRP) is a non-polar amino acid and is the only amino acid containing an indole ring: in addition to being involved in the biosynthesis and turnover of proteins and peptides, tryptophan (TRP) after its absorption into the body is converted into a series of small bioactive, pleiotropic compounds, each capable of influencing certain cellular metabolic pathways and physiological responses. Concerning these bio-transformations, L-TRP is processed through different pathways: 90–97% lead to the breakdown of the indole ring with the formation of kynurenine (KYN) and derivatives; 3–10% keep the indole ring intact and produce chemical messengers of the indolamine family, among which is melatonin (MEL) [1].

The KYN pathway is a metabolic route in which TRP is converted into compounds with different biological functions [2]. In humans, the KYN pathway is involved in neurodegenerative and autoimmune disorders, including Alzheimer’s, Huntington’s disease, amyotrophic lateral sclerosis diseases, and in cancer and psychiatric syndromes, including schizophrenia [3,4,5]. KYN itself is a ligand of the aryl receptor and therefore regulates gene expression and immune function [6,7]. The metabolites deriving from the KYN pathway are considered cytoprotective (kynurenic acid, KYNA) or cytotoxic/pro-epilepsy (3OH kynurenine, 3OH KYN, 3OH anthranilic acid, 3OH AA, quinolinic acid, and nicotinic acid). KYNA is an endogenous neuroprotector that is usually present in the brain in nanomolar concentrations. It is an antagonist of the nicotinic cholinergic receptor α7 and a non-competitive antagonist of ionotropic *N*-methyl-d-aspartate (NMDA) receptors [8], the activation of which would facilitate the initiation of the processes leading to the death of neurons after a short period of anoxia. KYNA has been shown to be the endogenous agonist of the GPR35 receptor expressed mainly in immune cells and peripheral monocyte/macrophage cells where it is able to reduce the secretion of tumor necrosis factor α (TNFα) induced by lipopolysaccharides [9]. It is an antioxidant and neuroprotective compound [10]; in particular, altered KYNA levels may suggest an inflammatory response, as shown in Alzheimer’s disease patients [11]. KYNA reduces the heart rate and glaucoma in mice and it has a role in carcinogenesis and cancer therapy [12,13,14].

Several studies have shown the existence of the KYN pathway in yeasts [15,16,17,18]. Panozzo and co-authors (2002) identified the genes that encode several KYN pathway enzymes in *Saccharomyces cerevisiae* [19]. Bna3p has been reported as a KYN aminotransferases (KAT) present in yeast, which converts KYN to KYNA [20]. According to Ohashi et al. (2017) [21], the KAT enzymes Aro8 and Aro9 reduce the toxic effects of some metabolites of TRP, allowing their conversion to less toxic metabolites for the cell. It has been shown that the biosynthesis of NAD^+^ and niacin through the KYN pathway is present in *S. cerevisiae* and *S. uvarum* [15,17]. Actually, the synthesis of NAD^+^ in yeasts occurs not only through de novo biosynthesis from TRP but also through the recovery pathway of the NAD^+^ precursors which are well-known vitamins such as nicotinic acid and nicotinamide. Furthermore, nicotinamide-ribose, nicotinamide mononucleotide, and nicotinic acid-ribose have been identified as NAD^+^ precursors [17,22,23,24]; they are transported into the yeast cells from the culture media and assimilated for the NAD^+^ supply [25,26]. KYNA production has been optimized in *Y. lipolytica* [27], an unconventional yeast with a high biotechnological potential. The results showed that *Y. lipolytica* S12 strain is able to produce KYNA in high concentrations (up to 21.38 μg/mL in culture broth and 494.16 μg/g cell dry weight in biomass) in optimized conditions in a medium supplemented with tryptophan (200 mg/L). Recently. Wrobel-Kwiatkowska et al. 2020 reported that KYNA may be efficiently produced by the yeast *Y. lipolytica* S12 in media containing chestnut honey up to 68 mg/L in culture broth and 542 mg/kg in yeast biomass [28]. KYNA release has been also reported in brewing experiments performed by *S. cerevisiae* and *Saccharomyces pastorianus* [18].

A little information is available on the biosynthesis of MEL in fungi; it can be synthesized by *S. cerevisiae* via two routes. Serotonin (5OH TRY) can be acetylated to N-acetyl serotonin (NAC 5OH TRY) or it can be converted to MEL through 5-methoxytryptamine (5OME TRY) [29,30]. To date, only the *PAA1* gene in *S. cerevisiae*, which codes for a polyamine acetyltransferase, has been proposed as a homologue of the *AANAT* coding for the enzyme arylalkylamine *N*-acetyltransferase present in vertebrates [31]. Kinetic studies have shown that this enzyme has a higher catalytic efficiency for 5OME TRY than 5OH TRY, suggesting that this could be the final enzyme in the MEL biosynthesis pathway. In another study, a different strategy was used to investigate the MEL production by *S. cerevisiae*. Through the addition of intermediates of the pathway in the cells, at different growth phases, intracellular and extracellular samples were analyzed to evaluate the presence of indole compounds. The data suggest that the first step of the pathway is the decarboxylation of TRP to tryptamine (TRY). TRY is then hydroxylated to 5OH TRY which is converted into NAC 5OH TRY or 5OME TRY [32]. This implies that *S. cerevisiae* may use more than one pathway for MEL biosynthesis. MEL, however, is not the only existing N-acetyl-methoxindolamine. Its structural isomers have also been discovered in the past 10 years. It has been calculated that 42 combinations could be possible considering the position of the two side chains connected to the indole ring. From the point of view of biological functions, the MEL isomers (MIs) show antioxidant and cytoprotective activity depending on the modification of the position of the two side chains in the indole ring [33,34,35]. In recent years, investigations regarding the beneficial effects of fermented foods on human health have increased considerably. This effect is often associated with the metabolism of microorganisms that, proliferating in different matrices, lead to the synthesis of neuroactive compounds [36]. As introduced above, food supplementation with dTRPs could be considered a strategy in the prevention of several diseases. However, dTRPs in natural sources are often in a little amount [37,38]. Thus, the natural enrichment in TRP of food raw materials potentially fermentable by selected dTRPs high-producing microorganisms represent a novel and feasible approach in the setup of novel functional foods. The present study aimed at evaluating and optimizing the release of dTRPs by *S. cerevisiae* EC1118 in laboratory cultural media. Then, dTRPs production was assessed in a medium naturally enriched in TRP by soybean addition with potential functional properties on human health. This last approach can be considered as a “case of study” since it cannot be compared with the current literature; indeed, no studies have investigated so far about the use of raw materials of food origin as a natural source of TRP aimed at obtaining dTRPs.

## 2. Results and Discussion

This section presents the results obtained from laboratory scale cultures of *S. cerevisiae* EC1118 aimed at evaluating the potential production of dTRPs both from the KYN pathway and, partially, from the serotonin and indole degradation routes. This yeast strain is a well-studied wine yeast strain showing a relevant fermentative fitness [39] and it was found to release dTRPs in a previous investigation [40]. After the assessment of the main compounds released in YNBT100 medium, the increase of the production of dTRPs was successfully reached by optimization of the cultural medium and through experiments of whole-cell bioconversion (WCB). Then, fermentation experiments in a medium with soy flour, naturally rich in TRP, were performed as case of study in order to assess the effects of yeast fermentation in the synthesis of dTRPs.

### 2.1. Quantitation of dTRPs during the Fermentation of S. Cerevisiae EC1118 in YNBT100 Medium

TRP is one of the most unstable amino acids and the two parameters that can mainly influence its stability are temperature and pH. In order to evaluate the thermal stability of a chemically defined medium containing 100 mg/L TRP, a preliminary test was carried out in YNBT100 in absence of inoculation applying the experimental conditions adopted in study (25 °C, static, aerobic). The initial concentration of TRP was estimated at 98.49 ± 3.16 mg/L. The concentration was detected at 101.19 mg/L ± 4.32 mg/L after 144 h from the beginning of the experiment, demonstrating that there was no significant degradation of TRP; therefore, a decrease of TRP content in subsequent trials must be exclusively linked to the metabolism of *S. cerevisiae* EC1118, employed as selected yeast in this work.

**Figure 1:**
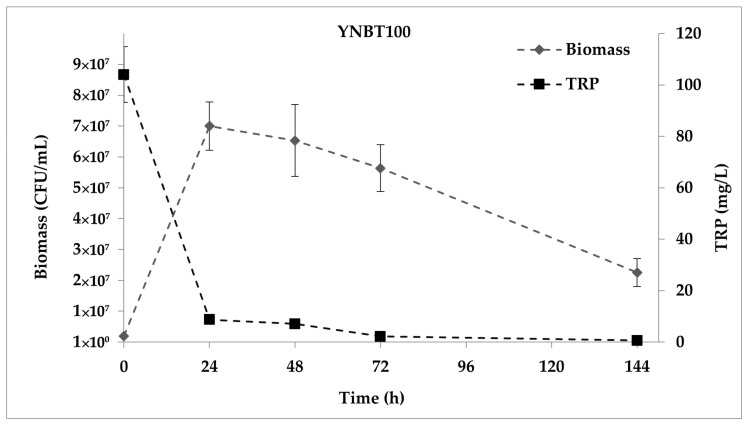
*S. cerevisiae* EC1118 growth curve in YNBT100 medium and TRP consumption.

The consumption of TRP and the cellular growth of *S. cerevisiae* EC1118 in YNBT100 medium during the fermentation is shown in Figure 1. The maximum level of yeast biomass was obtained after 24 h from the inoculum (7.00 ± 0.78 × 10^7^ CFU/mL); this level remained stable up to 144 h. At the end of the experiment, viable cells were reduced at 2.25 ± 0.46 × 10^7^ CFU/mL. The content of TRP at the starting time was 104.100 ± 10.800 mg/L. According to the yeast proliferation, its consumption mainly occurred in the first day of fermentation showing a 91.6% decrease. At 144 h from the inoculum, the TRP was almost depleted reaching a final amount of 0.549 ± 0.001 mg/L.

**Figure 2:**
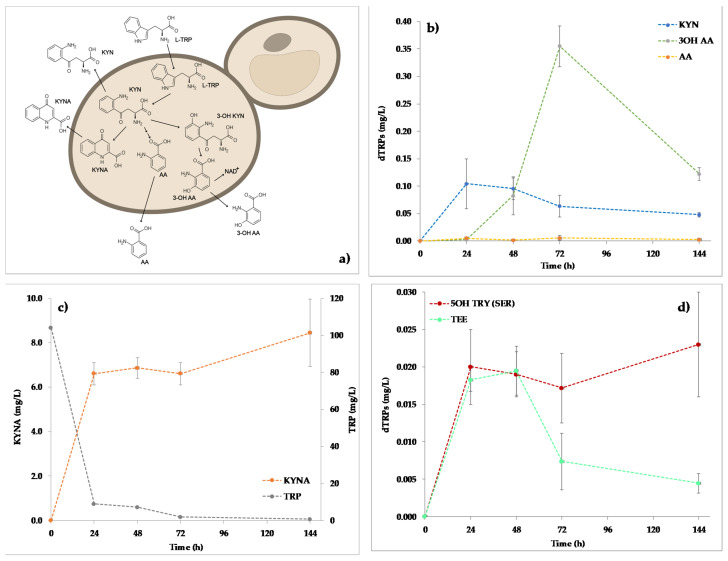
(**a**) Schematic view of the metabolites from the KYN pathway accumulated in YNBT100 medium and (**b**,**c**) kinetics production of the extracellular intermediates of the KYN pathway in *S. cerevisiae* EC1118. (**d**) Kinetics production of the extracellular intermediates of the MEL pathway in *S. cerevisiae* EC1118 in YNBT100.

The KYN pathway is an important metabolic pathway in which TRP is converted to compounds with different biological functions along three different branches (Figure 2a). The KYN route of the TRP degradation was followed throughout the kinetics production of TRP, KYN, 3OH KYN, KYNA, AA, and 3OH AA (Figure 2b).

Preliminary dosages demonstrated the absence of the analyzed molecules in the not inoculated cultural medium. Most of the assayed compounds increased accordingly with the yeast biomass level in the first 24 h after the inoculation. Indeed, KYN and KYNA accumulated in the supernatant up to 24 h (0.104 ± 0.046 and 6.603 ± 0.491 mg/L, respectively). Then, both compounds showed an accumulation plateau between 24–72 h. At 144 h, the trends of KYN and KYNA concentrations were opposite: while for KYN a significant decrease to 0.048 ± 0.040 mg/L (*p* < 0.05) was observed, KYNA conversely raised at 8.452 ± 1.516 mg/L. This finding confirms that in *S. cerevisiae* EC1118 the TRP consumption mainly leads to the breakdown of the indole ring with the formation of KYN and derivatives (KYN pathway) while a little proportion of reaction keep the indole ring intact and produce chemical messengers such as indolamines (serotonine and indole derivatives routes) [1]. Currently, there are no bibliographical references showing quantifications of KYN pathway metabolites by the yeast strain under study but Shin and collaborators (1991) [15] showed that *Saccharomyces uvarum* can potentially synthesize KYNA when TRP is present in the cultural medium. Recently, TRP and its derivatives were quantified in different beer samples and during the fermentation of wort by *S. cerevisiae* and *S. pastorianus* demonstrating that differences in KYN and KYNA levels could be related to the different fermentation conditions, process, and yeast strains [18]. In particular, *S. pastorianus* produced a smaller amount of the two compounds in comparison to *S. cerevisiae*. These results suggest that a significant portion of TRP is metabolized by the cells as a nitrogen source for the growth and the production of biomass; being an essential aromatic amino acid, TRP is used by yeast for the synthesis of proteins and other cellular compounds [41]. Also, the nitrogen requirement depends on the yeast species, explaining the observed difference in the consumption of TRP for *S. pastorianus* and *S. cerevisiae* [19,42]. Minor changes in AA production were detected; the evolution of this compound was quite stable during the experiment except for the sample point at 48 h where a significant but slight decrease of the molecule was found. On the contrary, a peak corresponding to the highest extracellular release of 3OH AA occurred at 72 h (0.355 ± 0.037 mg/L), when cells were in stationary phase. 3OH KYN failed to be accumulated in the medium.

Regarding the MEL biosynthesis pathway, 5OH TRP, serotonin (5OH TRY), NAC SER (NAC 5OH TRY), and MEL were measured, together with TEE (Figure 2c) and other potential MIs. Serotonin was the only intermediate detected. Its production trend was similar to the one of TEE up to 48 h. While the serotonin concentration was stable until the end of the trial, the TEE content decreased at the sampling time 72 h reaching its lowest value (between 0.004–0.007 mg/L). TEE was probably used by the yeast as a TRP source in the serotonin route since the amino acid in the medium was almost completely depleted already after 24 h from the beginning of the fermentation. 5OH TRP and NAC SER (NAC 5OH TRY) were never detected under the adopted experimental conditions. Moreover, unlike to what was observed for *S. cerevisiae* EC1118 [40], MEL levels were under the detection limit in the analyzed supernatants. This result could explain a poor technical caution in handling the samples that determined a rapid conversion of MEL to other indoles [43,44,45] or conversely, to confirm the already reported inconsistency in MEL release observed among biological replicates during yeast fermentations in standardized growth conditions [46]. However, the TRP metabolism of *S. cerevisiae* appears very sensitive to micro-environmental situation and physiological cell state. Indeed, Muniz-Calvo and collaborators (2019) were unable to detect melatonin formation in *S. cerevisiae* cultivated under different growth systems after tryptophan supplementation [32]; only by pulsing yeast cells with serotonin the melatonin production was observed. These results suggest that the TRP uptake and its further transformation to serotonin are heavily dependent by growth conditions and they are key elements in the MEL biosynthesis pathway.

**Figure 3:**
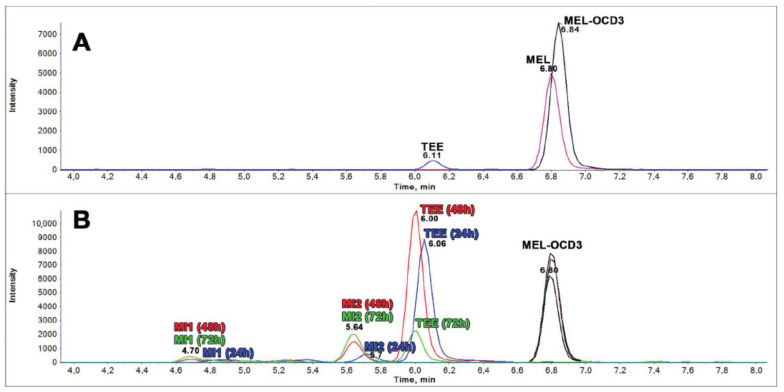
Melatonin isomers (MI) production by *S.cerevisiae* EC1118. Chromatogram in (**A**) shows the melatonin transition (*m/z* 233.2 > 174.2) of a pure standard containing melatonin (MEL, rt 6.80) tryptophan ethyl ester (TEE, rt 6.11) both at a concentration of 2 ng/mL and internal standard (MEL OCD3, rt 6.84, *m/z* 236.2 > 177.2, black). Chromatograms in (**B**) show the superimposition of melatonin trace (*m/z* 233.2 > 174.2) from three different *S. cerevisiae* EC1118 fermentation supernatants at 24 h (blue), 48 h (red), and 72 h (green). In (**B**) is evident the absence of the melatonin peak that should be expected at the same retention time of its labeled analogue (rt 6.80, black). Among melatonin isomers in fermentation medium it was evidenced the presence of tryptophan ethyl ester (TEE, rt 6.06) and two isomers, named MI1 (rt 4.70) and MI2 (rt 5.6), with a not-elucidated structure.

The release of two MIs at 4.7 min (MI1) and 5.6 min (MI2) retention times was measured (Figure 3). Literature reports that *S. cerevisiae* EC1118 is capable to produce MIs [40]; however, also their accumulation can significantly vary among the biological replicates or under different experimental conditions. In order to verify whether the formation of MIs was consistent in the biological replicates, three independent six-well plate yeast cultures of *S. cerevisiae* EC1118, prepared inoculating different pre-cultures obtained from different glycerol stocks, were monitored over the time. The analysis of variance and the grouping test of the mean values and the relevant sum of the two isomers showed differences statistically significant (*p* < 0.05) at the sampling times 24–48, 72, and 144 h (Table 1). This outcome suggests that MIs production is potentially dependent by the yeast growth phase and that, in YNBT100 medium, the higher release of MIs is obtained at 72–144 h. Contrarily, the ANOVA of the means of the ratio (MI1/MI2) resulted not significant along the sampling times (Table 1) indicating that the N-acetylserotonin methyltransferase enzyme, involved in the synthesis of MEL and its possible isomers, maintained a stable accumulation of the derived products (MI1 and MI2) during the yeast growth.

### 2.2. Production of dTRPs in YNBT400 Medium and in WCB Trials

In order to optimize the release of dTRPs by *S. cerevisiae* EC1118, fermentation and whole-cell biocatalysis processes were set up. In the first approach the growth test was performed in YNBT400 medium for 144 h, whereas in the second one the biotransformations were carried out in a buffer with TRP excess for 24 h. The comparison of these two approaches might be useful to assay the production of commercially valuable compounds that can be used in pharmaceutical and cosmetics fields or in the food and beverages sectors [47].

**Figure 4:**
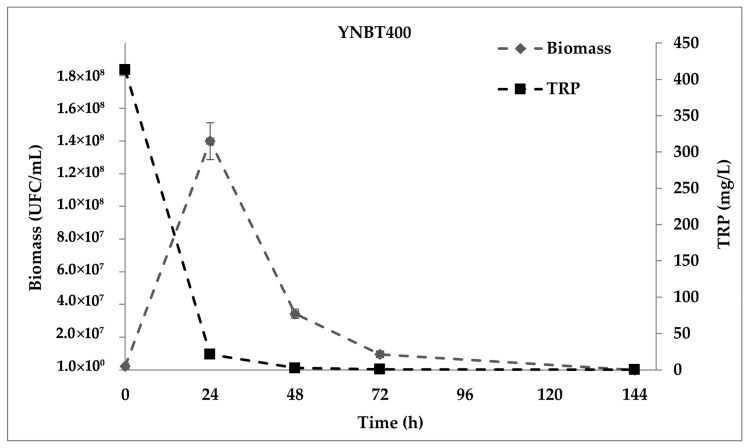
*S. cerevisiae* EC1118 growth curve in YNBT400 medium and TRP consumption.

As far the fermentations in YNBT400 medium, the cultural broth was prepared with 400 mg/L TRP and increasing the carbon and nitrogen sources, vitamins, salts, and trace elements. In general, the fermentation trend YNBT400 medium in 144 h was comparable to the one obtained in YNBT100 medium for the entire duration of the experiment (data not shown), showing a considerable increase of cells in the first 24 h; however, at this time point a two-fold increase in yeast cell count was registered (7.00 ± 0.07 × 10^7^ versus 1.40 ± 0.11 × 10^8^ CFU/mL in YNBT100 and YNBT400, respectively). At 48 h from the inoculation, glucose become a limiting factor for the yeast proliferation being almost depleted (0.0030 ± 0.0001 g/L). Accordingly, TRP consumption was rapid and it occurred almost completely within 48 h (Figure 4). However, the decrease of the cell viability did not correspond with a decrease in the optical density, which remained constant between 24–144 h (data not shown). This result suggests that cell autolysis did not occur under the investigated growth conditions.

**Figure 5:**
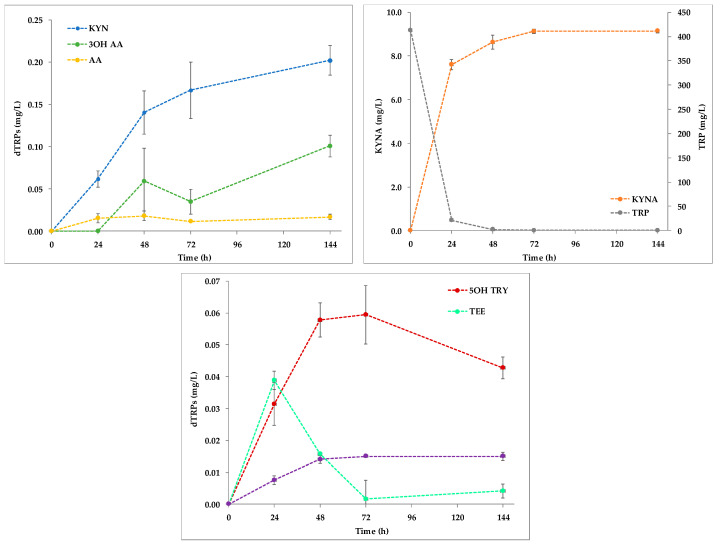
TRP derivatives produced by *S. cerevisiae* EC1118 in YNBT400 medium. Error bars indicate the standard deviation.

As described for the YNBT100 medium, a TRP catabolism oriented towards the KYN pathway was confirmed (Figure 5). Despite the biomass improvement, a significant increase in all the dTRPs analyzed in this study was not always obtained. While KYNA and 3OH AA (9.146 ± 0.585 and 0.101 ± 0.013 mg/L, respectively) reached concentrations comparable to the levels found in YNBT100 medium, four-fold higher KYN and MIs amounts were detected in YNBT400 cultures (Table 1).

**Figure 6:**
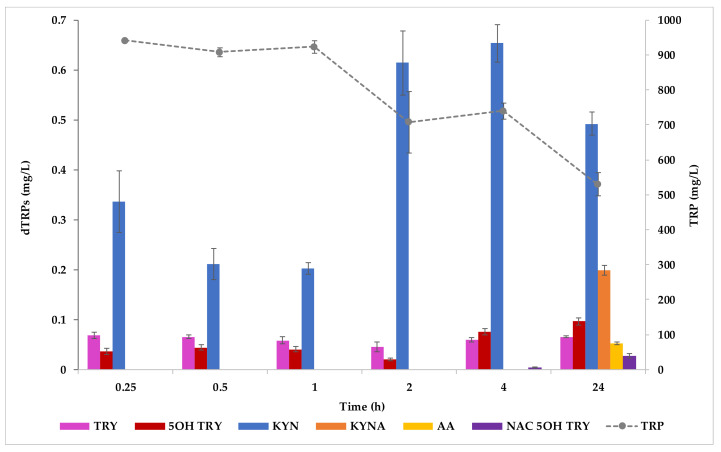
TRP derivatives produced by *S. cerevisiae* EC1118 in WCB experiments. Error bars indicate the standard deviation among the mean values of biological replicates.

Concerning the whole-cell biotransformation experiments, after yeast biomass was harvested and washed from pre-cultures grown in a rich medium, cells were suspended in the biocatalysis buffer containing 1 g/L TRP and glucose. The high TRP concentration aimed at forcing the production of dTRPs. The presence of glucose in the biotransformation medium represents an important element for the metabolic activity of cells since it helps cells restoring the redox potential, given by the NAD^+^/NADH ratio, and other cofactors such as ATP and those of the TCA cycle (i.e., 2-oxoglutarate, acetyl-CoA). After 24 h from the beginning of the WCB experiments glucose was depleted and a significant cell biomass increased was not detected (1.107 ± 0.012 OD_600/mL_ at the inoculum time versus 1.123 ± 0.017 OD_600/mL_ at 24 h). As observed in YNBT400 trials, cell autolysis did not occur (data not shown). TRP consumption was about 390 mg/L (from 923.41 ± 25.46 mg/L to 529.14 ± 46.57 mg/L); this amount was comparable with the one observed in fermentation tests in YNBT400 medium in 24 h, where about 410 mg/L TRP (from 413.32 ± 12.40 mg/L to 21.43 ± 0.047 mg/L) were used by cells. Only KYN, TRY, and 5OH TRY started to be accumulated immediately after 15 min from the beginning of the experiment, reaching a maximum concentration between 4 and 24 h of 0.653 ± 0.052 mg/L, 0.066 ± 0.001 mg/L, and 0.096 ± 0.010 mg/L, respectively (Figure 6).

The results confirm that *S. cerevisiae* EC1118 decarboxylates the TRP to TRY [32]. The extra and intracellular production of MEL and indoles in *S. cerevisiae* by adding intermediates to cells in different growth phases (exponential and stationary) was recently evaluated [32]; these authors revealed that serotonin mainly derived from the decarboxylation of TRP, followed by hydroxylation of TRY, as occurs in plants. Thus, MEL biosynthesis from serotonin can occur by N-acetylation followed by O-methylation or vice versa. KYNA, AA, NAC 5OH TRY, MI1, and MI2 were only detected at the end of the experiment (at 144 h) at 0.199 ± 0.014 mg/L, 0.052 ± 0.004 mg/L, 0.027 ± 0.006 mg/L, 0.201 ± 0.025 mg/L and 0.102 ± 0.016 mg/L, respectively. The synthesis of 5OH TRP, by TRP hydroxylase, was not detected. However, since 5OH TRP is an intermediate of the MEL biosynthesis pathway, further tests like a fluxomic analysis would be necessary to have more indications on its fate.

In order to directly compare the concentrations of the dTRPs synthesized in fermentations in YNBT400 and in WCB experiments, normalized data against the biomass levels and the fold change between the considered approaches were calculated (Table 2). This estimation was possible because at 24 h from the beginning of both processes the obtained dTRPs may potentially derive from a comparable TRP consumption (about 400 mg/L TRP, as an average). KYNA was the only compound that showed a higher concentration in fermentations in YNBT400 compared to the WCB tests (about 8 times). The other dTRPs gained higher concentrations in WCB experiments. Thus, depending on the compound a fermentation or a bioconversion can be settled; briefly, KYN increased 35 times, TRY 15 times, 5OH TRY 14 times, MI1 and MI2 15 and 24 times, respectively in bioconversion in respect to the fermentation (Table 2).

### 2.3. dTRPs from YNB-Soybean-Based Culture Medium

In order to evaluate the capability of *S. cerevisiae* EC1118 to release dTRPs in a food-based matrix naturally rich in TRP, an experiment in a soybean-based cultural medium (YNBSOY) was set up as a case of study. Nowadays, the beneficial effects on human health of several raw materials fermented throughout spontaneous or controlled processes has been proposed [48,49]. Thus, showing that the supply of dTRPs with diet—including KYNA, 5OH TRY, or MEL and its isomers—could be implemented by the consumption of fermented foods represents a health and marketing plus value concept. Vitalini and co-authors (2020) [50] have recently demonstrated that toasted soybean flour treated by a water extraction at room temperature allows to obtain free TRP potentially bioavailable during yeast fermentation as precursor of dTRPs. The controlled fermentation of the YNBSOY medium with *S. cerevisiae* EC1118 showed a high fermentative power; indeed, a weight loss of about 10 times higher was obtained at 72 h in comparison to the control (not inoculated). Contrarily to what was observed in previous fermentations in YNBT100 and YNBT400 media, the yeast growth increased up to the end of the trials. Glucose was completely consumed in 72 h (0.015 ± 0.009 g/L); however, in the control sample it decreased (from 47.632 ± 0.188 to 37.880 ± 1.126 g/L) indicating the presence of an indigenous soy flour microbiota that could trigger uncontrolled spontaneous fermentations. An increase in acetic acid concentration (from 0.126 ± 0.004 to 0.192 ± 0.001 g/L) was detected in the inoculated YNBSOY medium. Generally, *S. cerevisiae* is characterized by low production of acetic acid, with an average value of 300 mg/L [51]; the yeast under study, being an oenological strain, produces smaller quantities in the conditions under study. On the other hands, a more consistent increase in acetic acid concentration (0.302 ± 0.001 g/L), possibly attributable to the development of microorganisms already present in the flour, was observed in the control.

**Figure 7:**
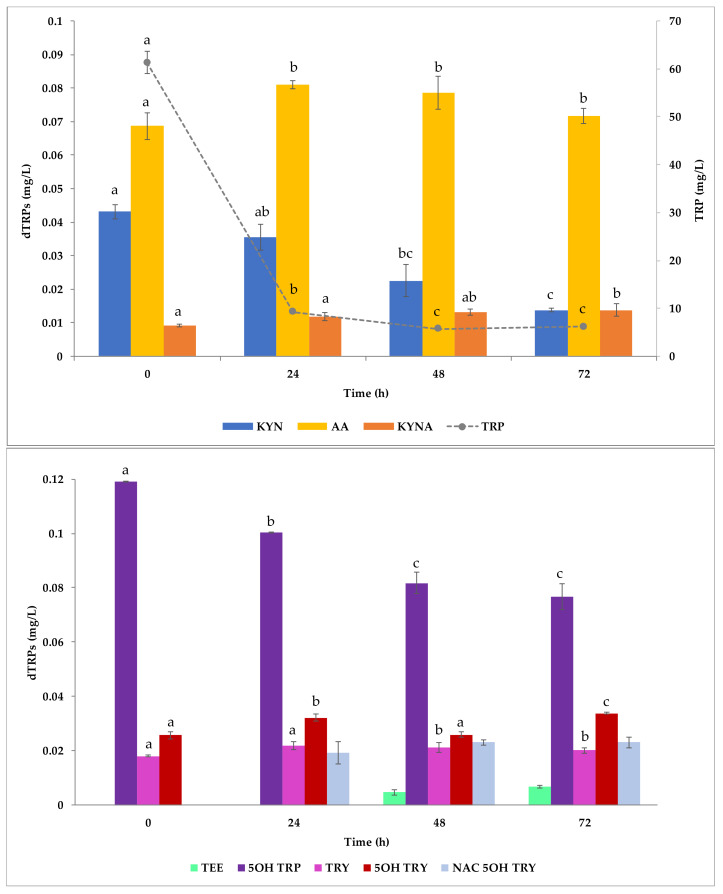
TRP derivatives produced by *S. cerevisiae* EC1118 in fermentation experiments in YNBSOY medium. Error bars indicate the standard deviation among the means of biological replicates. Letters indicate the grouping information using the Fisher LSD Method and 95% confidence.

Regarding the TRP available as substrate for indoles production, its initial concentration was detected at 61.301 ± 2.310 mg/L. Since YNBSOY is a YNB-based medium (already containing 20 mg/L TRP), it can be calculated that the soybean flour naturally enriched the growth culture of about 40 mg/L TRP. At the end of the experiment the TRP concentration was measured at 6.170 ± 0.003 mg/L. A preliminary investigation concerning the dTRPs content in YNBSOY medium revealed that some indoles were already present at the inoculum time; however, depending on the compound different fates were observed during the fermentation (Figure 7). While a decrease trend was found for KYN (from 0.039 ± 0.007 to 0.016 ± 0.004 mg/L), AA (from 0.027 ± 0.003 to 0.004 ± 0.001 mg/L), and 5OH TRP (0.090 ± 0.001 to 0.077 ± 0.005 mg/L), other compounds such as KYNA (from 0.009 ± 0.001 to 0.0137 ± 0.002 mg/L), TEE (from 0 to 0.006 ± 0.001 mg/L), and 5OH TRY (from 0.026 ± 0.001 to 0.036 ± 0.003 mg/L) increased. No significant variation of TRY and NAC 5OH TRY was observed. The two isomers, MI1 and MI2, detected in the fermentation trials in YNBT100 and YNBT400 media, failed to be released. Once again, the cultural conditions proved to be a key factor in the production of derivatives from the MEL biosynthesis pathway.

## 3. Materials and Methods

### 3.1. Yeast Strain

The yeast strain used in this work is *Saccharomyces cerevisiae* EC1118, commercial wine strain. The pure culture is maintained in YPD medium (1% yeast extract, 2% peptone, 2% glucose (*w*/*v*)) with 20% (*v*/*v*) glycerol at −80 °C.

### 3.2. Growth Conditions in YNB-Based Media

To evaluate the production of TRP derivatives (dTRPs) by *S. cerevisiae* EC1118, the strain was preliminary grown in a chemically defined cultural medium (20 g/L glucose; 6.7 g/L yeast nitrogen base (Difco, Heidelberg, Germany), pH 5.4) containing 20 mg/L TRP as a precursor (YNBT20). Then, the experiment was conducted in YNBT100 containing a final TRP concentration of 100 mg/L; to obtain this TRP amount, the amino acid was supplemented into YNBT20 medium as a stock solution (4 mg/mL) prepared in sterile demineralized water (sdH_2_O) and sterilized by filtration with 0.22 µm filters, immediately before inoculation. Growth tests were performed in sterile six-well plates with 10 mL of YNBT100 medium at 25 °C, 110 rpm Briefly, from the glycerol stock the yeast strain was preliminarily inoculated in three separated tubes containing 5 mL of YPD medium at 25 °C for 24 h to obtain biological replicates. From the three pre-cultures, cells in the exponential growth phase [about 2–4 OD_600nm_ measured in a UV–vis spectrophotometer, Jenway 7305, Stone, United Kingdom)] were inoculated at 1% (*v*/*v*) in three clean and sterile tubes containing 5 mL of YNB-based medium without amino acids [20 g/L glucose; 6.7 g/L YNB without amino acids, pH 5.4] at 25 °C for 72 h. Replicates were centrifuged at 4000 rpm (Hettich Zentrifugen, Rotina 380r, Tuttlingen, Germany) for 10 min, cells were washed with sdH_2_O and further centrifuged under the above-mentioned conditions. Each cell pellet was resuspended in three clean and sterile tubes containing 5 mL of YNB medium without amino acids and glucose [6.7 g/L YNB without amino acids, pH 5.4] for 24 h to allow nutrients depletion. Then, starved yeasts were centrifuged at 4000 rpm (Hettich Zentrifugen, Rotina 380r, Tuttlingen, Germany) for 10 min and resuspended in 2 mL of sdH_2_O. A 6-well plate was inoculated at 0.2 OD_600nm_ per well from each pre-culture. The plates were sealed with sterile parafilm to avoid any possible contamination. In addition, they were covered with aluminum foil to preserve the dTRPs from oxidative photodegration. The experiment lasted 144 h and the monitoring of the growth (by OD_600nm_ measurement and by cell plate counting) and the production of dTRPs was carried out at 0, 24, 48, 72, and 144 h after inoculation. Regarding the cell count, 100 µL of appropriate decimal serial dilutions in sdH_2_O water were spread on Petri dishes containing WL nutrient agar medium (Scharlab, Barcelona, Spain) and, after incubation at 25 °C for 3 days, the colonies were enumerated. For the quantification of dTRPs, 1 mL of culture was collected from two wells/plate, centrifuged at 10,000× *g* for 10 min and supernatants were 0.22 µm filtered and stored at −20 °C for further LC-MS/MS analysis. The analysis was carried out in triplicate on each biological replicate.

### 3.3. TRP Conversion Tests

A sterile six-well plate containing 10 mL of YNBT100 medium per well was placed at 25 °C, 110 rpm, the same experimental conditions further used in this study, for 6 days monitoring the content of TRP at 0, 24, 48, 72, and 144 h. The plate was sealed with sterile parafilm and covered with aluminum foil. One mL of medium was collected at each sampling time from three wells and the supernatant were 0.22 µm filtered and stored at −20 °C until to be subjected to the LC-MS/MS analysis.

### 3.4. Optimization of the Production of dTRPs: Approaches and Experimental Conditions

In order to increase the quantity of biomass produced and, consequently, the dTRPs formed by *S. cerevisiae* EC1118, two experiments were developed under different conditions: fermentation growth test in an optimized cultural medium (YNBT400) and whole-cell bioconversion (WCB) trials.

#### 3.4.1. YNBT400 Trials

All tests were prepared in sterile 250 mL flasks, closed with sterile cotton caps, containing 100 mL of YNBT400 medium (100 g/L glucose; 26.8 g/L yeast nitrogen base (Difco, Heidelberg, Germany), pH 5.4) containing 400 mg/L TRP as precursor of dTRPs. The yeast strain was first inoculated into YPD medium. Flasks were inoculated at 0.2 OD_600nm_ and incubated at 25 °C, 110 rpm for 6 days. Monitoring of cell growth was carried out at 0, 24, 48, 72, and 144 h by evaluating optical density at 600 nm (UV–vis spectrophotometer, Jenway 7305, Stone, United Kingdom) and plate count on WL nutrient agar medium. Residual glucose was determined with an enzymatic kit (K-FRUGL assay kit-Megazyme, Wicklow, Ireland). All tests were performed in triplicate.

#### 3.4.2. WCB Trials

Experiments were carried out to evaluate the capability of *S. cerevisiae* EC1118 to produce dTRPs working as a cell factory using TRP as a precursor. WCB trials were carried out with a total yeast biomass of 350 OD_600nm._ Three biological replicates were prepared in flasks at 25 °C, 110 rpm, in 300 mL bioconversion buffer containing 0.1 M phosphate buffer pH 6.7, 1 g/L TRP and 20 g/L glucose. Briefly, a pre-culture in 100 mL of YPD medium was obtained by inoculating the strain at 1% (*v*/*v*) from glycerol stocks and incubated for 24 h at 25 °C under stirring at 110 rpm. Then, cells were centrifuged at 4000 rpm (Hettich Zentrifugen, Rotina 380r, Tuttlingen, Germany) for 10 min, washed with sdH_2_O and further centrifuged under the above-mentioned conditions. The cell pellet was resuspended in 100 mL of YNB without amino acids and glucose, overnight incubated at 25 °C at 110 rpm for nutrient depletion. Then, biomass was spectrophotometrically determined (600 OD/mL) and a volume of cell suspension corresponding to a total biomass of 350 OD_600nm_ was centrifuged at 4000 rpm (Hettich Zentrifugen, Rotina 380r, Tuttlingen, Germany) for 10 min, cells were washed with 20 mL sdH_2_O, centrifuged again at the same above conditions and transferred to the bioconversion flask. The experiment lasted 24 h and the production of dTRPs was evaluated at 0, 15, and 30 min; and 1, 2, 4, and 24 h after the inoculation. One mL of culture was collected from the three biological replicates, centrifuged at 10,000× *g* for 10 min and the supernatants were 0.22 µm filtered and stored at −20 °C until to be subjected to the LC-MS/MS analysis. The final glucose concentration was measured via enzymatic kit (kit-Megazyme, Wicklow, Ireland).

### 3.5. TRP Extraction from Toasted Soybean

The organic soy flour was purchased from the “Fior di Loto” company (Turin, Italy). An aliquot of flour was placed in a fan oven for 24 h at 50 °C before extraction to eliminate as much water as possible. Different techniques were applied for the extraction of TRP from the food matrix, in order to compare their efficiency: Soxhlet extraction, methanol extraction at room temperature and extraction in water at room temperature [50]. All procedures were performed in low light conditions, to prevent degradation of metabolites. The extractions were set up in triplicates.

#### 3.5.1. Soxhlet Extraction

The extraction with Soxhlet apparatus was performed following two different procedures: (i) 21 grams of toasted soybean flour were first defatted with petroleum ether and dichloromethane and subsequently extracted with methanol; (ii) The soy flour was also subjected to extraction in Soxhlet directly in methanol. Each obtained extract was dried with a rotary evaporator, frozen at −80 °C overnight and freeze-dried for about 5 h. The lyophilized sample was then stored at 4 °C until analysis.

#### 3.5.2. Methanol Extraction at Room Temperature

A mixture of flour and methanol (1:10, *w*/*v*) was sonicated for 10 min, then stirred for 20 min (DLAB SK-O180-E). After decanting, the supernatant was centrifuged at 28,000× *g* for 10 min and filtered with 0.22 µm nylon filters. The extraction was repeated, and the combined supernatants were stored at −20 °C until analysis.

#### 3.5.3. Extraction in Water at Room Temperature

A mixture of flour and distilled water (1:10, *w*/*v*) was sonicated, then stirred for 20 min (DLAB SK-O180-E). After decanting, the supernatant was centrifuged at 28,000× *g* for 10 min and filtered with 0.22 µm nylon filters. The obtained sample was stored at −20 °C until analysis.

### 3.6. Fermentation Tests in Soybean Flour

A triplicate destructive test was set up in 100 mL sterile flasks containing 15 g of toasted organic soybean flour and 45 mL of YNBT20 medium added with 50 g/L of glucose (YNBSOY). Experiments were run at 25 °C for 72 h, in anerobiosis with GasPack jar system (Merck Millipore, Burlington, MA, USA). The yeast was preliminarily inoculated in YPD at 25 °C for 48 h. Each flask was inoculated at 0.2 OD_600nm_. Three non-inoculated flasks were also set up as a negative control. The microbial growth was monitored by flask weight loss due to CO_2_ loss and cell plate count at 0, 24, 48, and 72 h after inoculation. The flasks were weighed daily with a technical balance until a constant weight. Cell count was determined on WL agar medium after incubation at 25 °C for 2–3 days. At each sampling time, supernatants for the determination of glucose, acetic acid, and dTRPS was collected as well: 1 mL of culture was collected and centrifuged at 11,000× *g* for 15 min. The supernatants were 0.22 µm filtered and frozen at −20 °C. Glucose and acetic acid were determined with enzymatic kits (kit-Megazyme, Wicklow, Ireland). All tests were performed in triplicate. The production of dTRPs was measured by LC-MS/MS analysis.

### 3.7. Chemical Analysis of TRP Derivatives

Methanol and formic acid (FA) were purchased from Sigma-Aldrich (Milan, Italy). All the aqueous solutions were prepared using Milli-Q purified water (Millipore, Milan, Italy). The pure chemical standards (analytical grade) of TRY, NAC TRY, 5OH TRY, 5OME TRY, NAC 5OH TRY, 5F TRY, 5OH TRP, TRP, TRP D5, TEE, AA, 3OH AA, KYNA, KYN, 3OH KYN, and MEL were purchased by Sigma-Aldrich (St. Louis, MO, USA). The isotopic isomer MEL OCD3 was synthesized by Prof. Andrea Penoni (Department of Science and High Technology, University of Insubria, Varese, Italy).

#### 3.7.1. Purification of Supernatants Deriving from Yeast Fermentation

Supernatants deriving from fermentation (10 µL) were added to 50 µL of MIX IS (internal standards) (20 ng of TRP D5, 1 ng of 5F TRY and 1.1 ng of MEL OCD3) and 100 µL of precipitating solution (methanol + 0.5% FA). After stirring by vortexing and centrifugation 5 min at 12,000 rpm (Hettich Zentrifugen, MIKRO200, Tuttlingen, Germany), 100 µL were taken and dried under nitrogen. Subsequently, the sample was resuspended with 50 µL of dH2O + 0.5% FA and 10 µL were injected directly into LC-MS/MS.

#### 3.7.2. Selective Extraction of Melatonin from Yeast Fermentation

Supernatants deriving from the fermentations (500 µL) were added to 50 µL of MIX IS; subsequently, they were purified by solid-phase extraction (SPE) with Strata X-Polymeric Reversed Phase 30 mg/1 mL (Phenomenex, Torrance, CA, USA) [52]. Before use, the cartridges were conditioned with 1 mL of methanol and 1 mL of dH_2_O. The samples, diluted with water to 1 mL as final volume, were loaded into the cartridges and then washed with 1 mL of water and 1 mL of 5% MeOH (*v*/*v*). After 5 min of vacuum drying to remove the excess of water, the residual compounds were eluted with 1 mL of methanol. The eluate was dried by nitrogen, the residue was resuspended with 100 µL of dH_2_O + 0.5% FA and 10 µL was injected for LC-MS/MS analysis.

#### 3.7.3. LC-MS/MS Analysis for the Detection of dTRPs

The analytical system consisted of an HPLC coupled to a tandem mass spectrometer, specifically a Dionex 3000 UltiMate instrument (Thermo Fisher Scientific, MA, USA) coupled to a tandem mass spectrometer (AB Sciex 3200 QTRAP, Concord, ON, Canada). The separation was obtained on reverse phase Zorbax Eclipse XDB-C18 (4.5 × 50 mm, 1.8 µm). The linear gradient was obtained by mixing the eluent A (dH_2_O + 0.1% of formic acid) and eluent B (methanol + 0.1% of formic acid). The elution gradient was set as follows: 0–1 min (20% B), 1–5 min (20–60% B), 5–7 min (60% B), 7.0–7.2 min (60–95% B), 7.2–8.2 min (95% B), 8.2–8.5 min (95–5% B), 10 (5% B). The flow rate was 0.4 mL/min, and the column temperature 40 °C. Multiple reaction monitoring (MRM) mode was used in electrospray positive mode (Table A1, Appendix A). A quantitative analysis was performed interpolating each area of the analyte peak/IS area with the calibration curve of each derivative of the TRP. Further analytical details can be found in [50].

#### 3.7.4. Validation of the Analytical Method

The method performances (specificity, precision, accuracy, linearity, the limit of detection (LOD) and limit of quantification (LOQ)) were reported in full details elsewhere [50]. The LOQ (the lowest concentration that produces a signal-to-noise ratio greater than 10) of analytes extracted from medium following the described procedure ranged from 2–50 ng/mL. The performance of melatonin extracted by SPE was studied in a range of 2–0.25 ng/mL and resulted to be acceptable for our purposes (linearity equation y = 0.7582× −0.0062, coefficient of determination R^2^ 0.994, LOQ 50 pg/mL and LOD 20 pg/mL).

### 3.8. Statistical Analysis

Where indicated the results were reported as mean ± standard deviation. Significant differences (*p* < 0.05) were evaluated by analysis of variance (one-way ANOVA) by using MINITAB Statistical Software 19 (Pennsylvania State University, PA, USA). Grouping information was determined using the Fisher LSD Method and 95% confidence.

## 4. Conclusions

Thanks to the advent of microbiological and chemical cutting-edge researches, many of the health benefits attributed to fermented foods and beverages are nowadays recognized. In particular, omics studies of traditional fermented foods have highlighted the microbiota of several spontaneous fermentations, revealing its role in the production of bioactive molecules [48]. The involvement of yeasts in the fermentation of several raw materials is well recognized in numerous productions, starting from the manufacture of alcoholic beverages (wine, beer, cider, and fruit wines) to fermented cereal-based doughs, and dairy, meat and fish products [53]. Yeasts share with animals several metabolic pathways which are relevant reservoirs of functional molecules, TRP derivatives above all [49]. The present study revealed the presence of variable amounts of dTRPs with a prevalence of metabolites of the KYN pathway, emphasizing that TRP metabolism of *S. cerevisiae* EC1118 pushed in this direction. The LC-MS/MS analysis showed that the compound synthesized in higher concentrations is KYNA, a molecule with neuroprotective and antioxidant properties. The fermentation in YNBT400 resulted the most promising approach for KYNA production which reached the highest concentrations (9.146 ± 0.585 mg/L). In comparison to literature data, the maximum concentration of KYNA measured in the post-culture medium was higher than KYNA levels in plasma of healthy subjects (5.8 ng/mL) [54] and milk of healthy breast-feeding women (3.9–56.6 microg/L) [55]. In addition, KYNA was detected in artificial baby formulas at lower concentrations (5.0–7.3 microg/L) [55]. Therefore, the supraphysiological levels of KYNA found in our experiments may exert therapeutic effects in human, even if the latter were mainly investigated in patients with neurological and psychiatric disorders in relation to brain and cerebrospinal fluid KYNA [56]. It is worth noting that the supplementation of rat maternal diet with KYNA in drinking water resulted in its increase in milk harvested, and the rat offspring fed with breast milk with artificially enhanced KYNA content demonstrated a lower mass gain during the first 21 days of life, which indicates that KYNA may act as an anti-obesity agent, as demonstrated from the body composition analysis [55]. Although the KYNA obtained in this way should be purified from the medium, probably with high costs compared to the organic synthesis, this work highlights the chance to obtain functional foods enriched with molecules with nutritional or therapeutic potential throughout the use of natural biological systems. Cells, for example, could be naturally oriented towards the production of specific dTRPs by enriching the cultures with more specific precursors other than TRP. However, the feasibility of the method proposed as an alternative approach of obtaining bioactive metabolites using biological systems needs to be evaluated, in terms of costs, instruments, time required, and yield.

As far MEL, detectable amounts were not measured in our experimental conditions (LOQ 50 pg/mL and LOD 20 pg/mL). However, two MEL isomers were successfully detected both in fermentation in YNBT400 and WCB experiments. In general, since the production of dTRPs could be a species- and strain-specific character [40] a full screening of dTRPs capable yeasts from wine, brewer and distillery should be performed in future investigations.

In conclusion, considering the high interest toward the dTRPs in the panorama of functional foods, the results of the present research provide the basis for in-depth studies on the effects of fermentation in the synthesis of compounds with important bioactive properties in novel food products.

## Figures and Tables

**Table 1:** MI1 and MI2 productions. Letters indicate different significant values (*p* < 0.05) at the yeast growth phases (24, 48, 72, and 144 h). Concentrations of MIs and their sums are here expressed in μg/L, considering the melatonin response. Letters indicate the grouping information using the Fisher LSD Method and 95% confidence.

Time (h)	YNBT100				YNBT400			
	MI1	MI2	Sum	MI1/MI2	MI1	MI2	Sum	MI1/MI2
24	14.6 ± 1.7 ^a^	6.9 ± 1.0 ^a^	21.8 ± 1.5 ^a^	2.2 ± 0.4	54.8 ± 1.4 ^a^	24.3 ± 6.2 ^a^	79.1 ± 4.9 ^a^	2.4 ± 0.7
48	14.2 ± 2.0 ^a^	6.4 ± 1.5 ^a^	20.6 ± 3.0 ^a^	2.3 ± 0.4	99.5 ± 5.0 ^b^	42.3 ± 6.0 ^b^	141.8 ± 1.3 ^b^	2.4 ± 0.4
72	20.4 ± 2.7 ^b^	10.4 ± 1.6 ^b^	30.7 ± 3.8 ^b^	2.0 ± 0.3	109.5 ± 2.5 ^c^	44.7 ± 3.7 ^b^	154.2 ± 6.0 ^c^	2.5 ± 0.2
144	27.8 ± 1.7 ^c^	11.2 ± 1.2 ^b^	38.9 ± 2.3 ^c^	2.5 ± 0.3	106.2 ± 8.5 ^bc^	37.5 ± 1.8 ^b^	143.6 ± 9.9 ^b,c^	2.8 ± 0.2

**Table 2:** Comparison of the production of dTRPs in fermentations in YNBT400 medium and in WCB experiments at 24 h. The productions were normalized against the yeast biomass and expressed as mg L^−1^ 10^−8^ cells. The fold change of the main dTRPs was calculated as the ratio between the concentration of the corresponding metabolite in the two investigated approaches.

dTRPs	YNBT400 (mg L^−1^ 10^−8^ Cells)	WCB (mg L^−1^ 10^−8^ Cells)	Fold Change
KYNA	5.449 ± 0.276	0.622 ± 0.000	8.76
KYN	0.044 ± 0.003	1.538 ± 0.008	0.029
TRY	0.014 ± 0.004	0.206 ± 0.010	0.068
5OH TRY	0.022 ± 0.003	0.301 ± 0.008	0.073
MI1	0.040 ± 0.003	0.626 ± 0.035	0.064
MI2	0.015 ± 0.003	0.354 ± 0.024	0.042

## Data Availability

Not applicable.

## Abbreviations

TRY	Tryptamine
NAC TRY	N-Ac Tryptamine
5OH TRY	5-OH Tryptamine or Serotonin
5OME TRY	5-OCH_3_ Tryptamine
NAC 5OH TRY	N-Ac-5-OH Tryptamine
5F TRY; IS1	5-F Tryptamine
5OH TRP	5-OH Tryptophan
TRP	Tryptophan
TRP D5; IS2	Tryptophan D_5_
TEE	Tryptophan Ethyl Ester
AA	Anthranilic acid
3OH AA	3-OH Anthranilic acid
KYNA	Kynurenic acid
KYN	Kynurenine
3OH KYN	3-OH Kynurenine
MEL	Melatonin or N-Ac-5- OCH3 Tryptamine
MEL OCD3, IS3	Melatonin OCD_3_
SPE	Solid Phase Extraction

## Appendix A

**Table A1:** MRM transitions for LC-MS/MS analysis of indolic species. Each subclass is preceded by the correspondent IS (in italics) used for quantification.

Name	MW	Q1	Q3	DP	CE	Rt
*5F TRY(IS1)*	*178*	*179.2*	*162.2*	*20*	*15.2*	*3.89*
TRY	160	161.2	144.2	20	14.8	2.89
5OH TRY (or Ser)	176	177.2	160.2	20	14.7	1.30
5OCH3 TRY	190	191.2	174.2	20	14.3	3.13
NAc TRY	202	203.2	144.2	20	19.1	6.87
NAc 5OHTRY (or N-Ac SER)	218	219.2	160.2	20	18.5	3.71
*TRP D5 (IS2)*	*209*	*210.2*	*192.2*	*20*	*14.8*	*3.03*
3OH AA	153	154.1	135.9	21	17.0	2.91
3OH KYN	224	225.1	208.1	16	13.0	1.30
AA	137	138.1	119.8	21	15.0	5.63
KYNA	189	190.1	89.1	36	51.0	4.56
KYN	208	209.2	192.2	21	15.0	1.72
TRP	204	205.2	188.2	20	13.4	3.12
TEE	232	233.2	216.2	20	13.1	6.06
5OH TRP	220	221.1	204.2	16	15.0	1.60
*MEL OCD3 (IS3)*	*235*	*236.2*	*177.2*	*20*	*19.1*	*6.80*
MEL	232	233.2	174.2	20	18.8	6.84

MW: molecular weight, Q1: molecular ion, Q3: daughter ion, DP: declustering potential, CE: collision energy, Rt: retention time.
